# Outcome of microscopically non-radical oesophagectomy for oesophageal and oesophagogastric junctional cancer: nationwide cohort study

**DOI:** 10.1093/bjsopen/zrab038

**Published:** 2021-05-11

**Authors:** P Hollertz, M Lindblad, P Sandström, I Halldestam, D Edholm

**Affiliations:** 1 Department of Surgery, Biomedical and Clinical Sciences, Linköping University, Linköping, Sweden; 2 Department of Surgery, Västervik Hospital, Västervik, Sweden; 3 Division of Surgery, Centre for Digestive Diseases, Karolinska University Hospital, Stockholm, Sweden; 4 Department of Clinical Science, Intervention and Technology, Karolinska Institutet, Stockholm, Sweden

## Abstract

**Background:**

Microscopically non-radical (R1) oesophageal cancer resection has been associated with worse survival. The aim of this study was to identify risk factors for R1 resection and to investigate how this affects long-term survival.

**Methods:**

The Swedish National Register for Oesophageal and Gastric Cancer was used to identify all patients who underwent oesophageal cancer resection with curative intent between 2006 and 2017. Risk factors for R1 resection were assessed by multivariable logistic regression analysis, and factors predicting 5-year survival identified by multivariable Cox regression.

**Results:**

The study included 1460 patients. Surgical margins were involved microscopically in 142 patients (9.7 per cent). The circumferential resection margin was involved in 114 (7.8 per cent), the proximal margin in 53 (3.6 per cent), and the distal margin in 29 (2.0 per cent). In 30 specimens (2.1 per cent), two or all three margins were involved. Independent risk factors for R1 resection were male sex, low BMI, absence of neoadjuvant treatments, and clinical T4 disease. The 5-year survival rate for the entire cohort was 42.2 per cent, but only 18.0 per cent for those who had an R1 resection. Independent risk factors for death within 5 years of resection were male sex, age above 60 years, normal BMI, ASA fitness grade III, intermediate-level education, R1 resection (hazard ratio 1.80, 95 per cent c.i. 1.40 to 2.32), clinical T3 disease, and clinical lymph node metastasis.

**Conclusion:**

R1 resection is common and predicts poor 5-year survival. Absence of neoadjuvant treatment is a risk factor for R1 resection.

## Teaser

Data from Swedish National Register for Oesophageal and Gastric Cancer were used to study risk factors for R1 resection, and to investigate how R1 affects long-term survival. R1 resection is common and predicts poor 5-year survival. Absence of neoadjuvant treatment and advanced tumour stage are risk factors for R1 resection.

## Introduction

The incidence of cancer of the oesophagus and oesophagogastric junction is increasing in many populations^1^. Most patients are not eligible for treatment with curative intent because they have metastatic disease or co-morbidities, so this cancer is the seventh leading cause of cancer deaths, with around 250 000 annually^1^.

Median survival is approximately 48 months for fit patients with localized disease who undergo chemoradiotherapy followed by *enbloc* resection of the oesophagus and locoregional lymph nodes^2^. The prognosis is highly dependent on clinical stage, particularly the presence of lymph node spread. The 5-year survival rate is 45–50 per cent in patients with node-negative oesophageal adenocarcinoma compared with 30 per cent in those with node-positive disease^3^.

Prognosis is also influenced by the extent of locoregional tumour clearance. Microscopic non-radical resection (R1), where cancer cells are identified at the resection margin, is an adverse prognostic factor compared with tumour-free margins (R0). It is unclear whether this worse prognosis is due to the inadequate surgical margin or whether R1 is a marker of more aggressive tumour biology. A correlation between positive resection margin and locoregional lymph node metastasis with advanced tumour stage has been demonstrated previously^4^^,^^5^. Some studies have identified positive margin as an independent negative prognostic factor for survival after resection for cancer of the oesophagus and oesophagogastric junction^4^, whereas others have not^6^.

Many studies looking at the influence of R status have been small^7^ and definitions of R1 resection rely on two different systems. The UK Royal College of Pathologists^8^ definition includes resections with tumour cells identified within 1 mm of a margin as R1, whereas the College of American Pathologists (CAP)^9^ requires tumour cells to be present at a margin. Some studies have focused on the importance of a positive circumferential margin^6^. Preoperative factors previously demonstrated to increase the risk of R1 resection include tumour location in the upper third of the oesophagus, tumour of T3 grade or above, and malnutrition, whereas neoadjuvant therapy, in particular chemoradiotherapy, decreases the risk of finding cancer cells at the resection margin^4^^,^^10^. Although some have found no difference in preoperative factors leading to R0 *versus* R1 status^11^, others have suggested that advanced clinical tumour category and low annual centre caseload might increase the risk of failing to achieve an R0 resection^12^.

The aim of this study was to determine risk factors for R1 resection using the CAP definition to see how R1 affects 5-year survival in a national cohort.

## Methods

###  

This was a register-based cohort study of all patients in Sweden who underwent curative resection for cancer of the oesophagus or oesophagogastric junction between 2006 and 2017. Risk factors for R1 resection were identified and the impact of R1 resection on survival assessed. Ethical permission was received from the Stockholm Regional Ethical Board (2013/596–31/3). The study was not preregistered.

### Study population

Patients were identified in the Swedish National Register for Oesophageal and Gastric Cancers (NREV), which has previously been shown to correctly identify 96 per cent of resections performed in Sweden^13^. Patients who had an elective resection of an oesophageal or oesophagogastric junctional cancer were included. Those having endoscopic, emergency, palliative or macroscopically non-radical resection (R2), and those for whom the resection specimen pathology report was missing, were excluded.

### Exposure and outcome data

R1 resection was defined by the identification of cancer cells at any of the longitudinal (proximal or distal) or the circumferential margins in accordance with CAP criteria^9^. NREV data were cross-matched by individual linking using Personal Registration Numbers with the National Inpatient and Outpatient Register, Emigration Register, and Death Register to obtain exposure data on potential risk factors and confounding variables. Patients were followed until death, emigration or the end of study on 30 November 2018, with a minimum follow-up of 12 months, whichever came first. Dates of death and emigration were obtained by cross-matching data from the NREV with those in the Total Population Register. Overall survival time was measured from the date of referral until the end of follow-up.

### Statistical analysis

The relationship between characteristics of patients who had R1 *versus* R0 resection was examined by univariable analysis, using the Mann–Whitney *U* test for continuous variables and χ^2^ test for categorical data. The following variables were explored: sex, age (categorized as under 60, 60–74, 75 years or older), BMI (below 18.5, 18.5–25.0, at least 25 kg/m^2^), WHO performance score (0–1, 2 or more), ASA fitness grade (I, II, III, IV), level of education (9 years or less—low, 10–12 years—intermediate, more than 12 years—high), preoperative fluorodeoxyglucose (FDG) PET–CT (yes, no), neoadjuvant treatment (chemoradiotherapy, chemotherapy, radiotherapy, none), surgical technique (2-phase, 3-phase, transhiatal), time period in which surgery was performed (2006–2011, 2012–2017), annual oesophagectomy caseload at centre (more than 20, 10–20, 2–10, less than 2 per year), clinical tumour category (cT1, cT2, cT3, cT4), clinical node status (cN0, cN1–3), tumour location (proximal third, middle third, lower third, oesophagogastric junction), histopathology (oesophageal adenocarcinoma, squamous cell carcinoma, undifferentiated), and lymph node harvest (continuous variable). Clinical TNM status was established after preoperative imaging according to the eighth edition of the TNM classification^14^. Pathological tumour category (pT) was established after analysis of the resection specimen. The risk of R1 resection was estimated with adjustment in multivariable logistic regression analysis; odds ratios (ORs) with 95 per cent confidence intervals were calculated. To assess the impact of R1 status on survival, a multivariable Cox regression analysis was undertaken, including variables potentially affecting survival, with calculation of hazard ratios (HRs) and 95 per cent confidence intervals. To avoid confounding, variables with *P* < 0.100 in univariable analysis were included in the multivariable models. Missing data for any of the parameters resulted in exclusion of that record from the corresponding univariable analysis and the multivariable analysis. Statistical analysis was done using Stata^®^/IC 15.1 SE (StataCorp, College Station, Texas, USA).

## Results

**Fig. 1:**
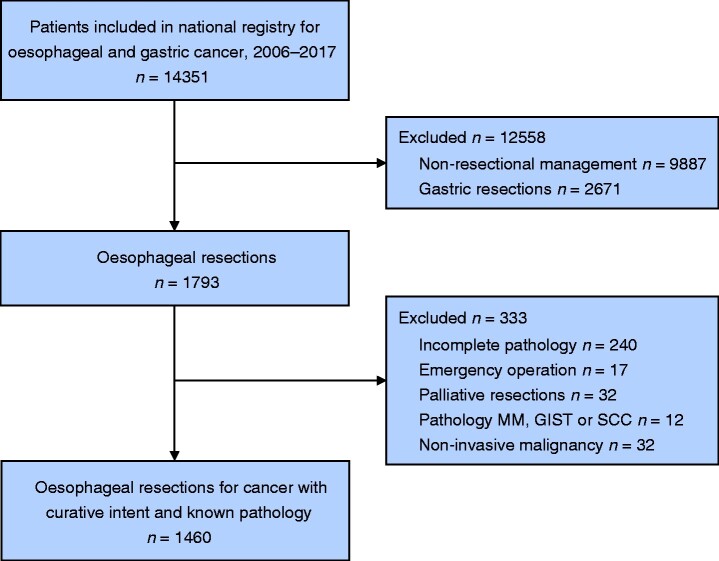
Study flow diagram MM, malignant melanoma; GIST, gastrointestinal stromal cell tumour; SCC, small cell carcinoma.

In all, 1460 patients who underwent resection of an oesophageal or oesophagogastric junctional cancer were included in the final analysis (*Fig. 1*). Mean and median follow-up were 47 and 32 months respectively.

Characteristics of the study population are presented in *Table 1*. The median age at diagnosis was 65 (range 22–87) years, and 79.8 per cent were men. Of all patients, 53.3 per cent had cT3 or cT4 tumours, 42.2 per cent had locoregional lymph node metastasis, and more than 80 per cent had adenocarcinomas. Nearly two-thirds of all patients received neoadjuvant therapy.

**Table 1:** Characteristics of study cohort

	All patients (*n* = 1460)		R0 (*n* = 1318)			R1 (*n* = 142)	*P* ^‡^
**Age (years)**							0.619
<60	399 (27.3)		360 (90.2)			39 (9.8)	
60–74	841 (57.4)		763 (90.9)			78 (9.1)	
≥75	220 (15.1)		195 (88.6)			25 (11.4)	
**Sex ratio (M : F)**	1165 : 295		1039 : 279			126 : 16	0.005
**BMI (kg/m^2^)**							0.004
<18.5	48 (3.3)		39 (81)			9 (19)	
18.5–25.0	589 (40.3)		519 (88.1)			70 (11.9)	
>25.0	754 (51.6)		696 (92.3)			58 (7.7)	
Missing	69 (4.7)		64 (93)			5 (7)	
**WHO performance score**							0.032
0–1	1390 (95.2)		1260 (90.7)			130 (9.3)	
>1	70 (4.8)		58 (83)			12 (17)	
**ASA fitness grade**							0.009
I	519 (35.5)		484 (93.3)			35 (6.7)	
II	707 (48.4)		630 (89.1)			77 (10.9)	
III	200 (13.7)		174 (87.0)			26 (13.0)	
IV	7 (0.5)		5 (71)			2 (29)	
Missing	27 (1.8)		25 (93)			2 (7)	
**Level of education (years)** ^†^							0.269
Low (≤9)	483 (33.1)		429 (88.8)			54 (11.2)	
Intermediate (10–12)	642 (44.0)		583 (90.8)			59 (9.2)	
High (>12)	318 (21.8)		293 (92.1)			25 (7.9)	
Missing	17 (1.2)		13 (76)			4 (24)	
**Preoperative FDG-PET–CT**							0.500
Yes	395 (27.1)		360 (91.1)			35 (8.9)	
No	1065 (72.9)		958 (90.0)			107 (10.0)	
**Neoadjuvant therapy**							<0.001
Chemoradiotherapy	583 (39.9)		549 (94.2)			34 (5.8)	
Chemotherapy	338 (23.2)		303 (89.6)			35 (10.4)	
Radiotherapy	5 (0.3)		5 (100)			0 (0)	
None	534 (36.6)		461 (86.3)			73 (13.7)	
**Surgical technique**							0.100
2-field resection	1147 (78.6)		1041 (90.8)			106 (9.2)	
3-field resection	162 (11.1)		148 (91.4)			14 (8.6)	
Transhiatal	151 (10.3)		129 (85.4)			22 (14.6)	
**Year of surgery**							0.120
2006–2011	701 (48.0)		624 (89.0)			77 (11.0)	
2012–2017	759 (52.0)		694 (91.4)			65 (8.6)	
**Centre caseload (per year)**							0.015
>20	570 (39.0)		509 (89.3)			61 (10.7)	
10–20	520 (35.6)		484 (93.1)			36 (6.9)	
2–10	250 (17.1)		224 (89.6)			26 (10.4)	
<2	120 (8.2)		101 (84.2)			19 (15.8)	
**Clinical tumour category**							0.009
cT1	93 (6.4)		88 (95)			5 (5)	
cT2	430 (29.5)		402 (93.5)			28 (6.5)	
cT3	717 (49.1)		634 (88.4)			83 (11.6)	
cT4	62 (4.2)		53 (85)			9 (15)	
Missing	158 (10.8)		141 (89.2)			17 (10.8)	
**Clinical lymph node status**							0.904
Negative	802 (54.9)		728 (90.8)			74 (9.2)	
Positive	616 (42.2)		558 (90.6)			58 (9.4)	
Missing	42 (2.9)		32 (76)			10 (24)	
**Tumour location**							0.440
Proximal third oesophagus	28 (1.9)		26 (93)			2 (7)	
Middle third oesophagus	182 (12.5)		170 (93.4)			12 (6.6)	
Lower third oesophagus	539 (36.9)		482 (89.4)			57 (10.6)	
Oesophagogastric junction	646 (44.3)		585 (90.6)			61 (9.4)	
Missing	65 (4.5)		55 (85)			10 (15)	
**Tumour histology**							0.871
Adenocarcinoma	1195 (81.2)		1077 (90.1)			118 (9.9)	
Squamous cell carcinoma	257 (17.6)		234 (91.1)			23 (9.0)	
Undifferentiated	8 (0.6)		7 (88)			1 (12)	
Lymph node yield*	20 (0–99)		20 (0–99)			18 (0–62)	0.116^§^

Values in parentheses are percentages unless indicated otherwise; *values are median (range). FDG, fluorodeoxyglucose. ^†^Low, corresponds to International Standard Classification of Education (ISCED) level 0–2; intermediate, corresponds to ISCED level 3; high, corresponds to ISCED level 4–6. ^‡^c^2^ test, except ^§^Mann–Whitney *U* test.

### Risk factors for microscopic tumour involvement of surgical margin (R1 resection)

R1 resection was confirmed in 142 specimens (9.7 per cent). Of these, the circumferential margin was involved in 114 (7.8 per cent), the proximal margin in 53 (3.6 per cent), and the distal margin in 29 (2.0 per cent). Thirty patients (2.1 per cent) had two or all three involved margins.

Compared with patients who had R0 resection, univariable analysis indicated that those who underwent R1 procedures were more likely to be men than women (10.8 *versus* 5.4 per cent; *P* = 0.005), underweight than normal weight or overweight (18.8 *versus* 11.9 and 7.7 per cent; *P* = 0.004), and to have more advanced clinical tumour stages (5.4 per cent cT1, 6.5 per cent cT2, 11.6 per cent cT3, and 15 per cent cT4; *P* = 0.009). R1 resection was more common with a transhiatal technique than with two- or three-field open resections, although these differences were not significant (14.6 *versus* 9.2 and 8.6 per cent; *P* = 0.100). R1 resection was more common in patients receiving neoadjuvant chemotherapy or no neoadjuvant treatment than among those who had neoadjuvant chemoradiotherapy (10.4 and 13.7 per cent *versus* 5.8 per cent; *P* < 0.001). There was no significant difference in R1 frequency with regard to age, level of education, use of preoperative FDG-PET–CT, year of surgery, clinical lymph node status, tumour location, tumour histology or lymph node yield (*Table 1*).

Multivariable logistic regression analysis showed an independently increased risk of R1 resection among men (OR 2.50, 95 per cent c.i. 1.36 to 4.58), those who were underweight (OR 2.75, 1.10 to 6.83), and patients with advanced tumour stage (cT4: OR 3.65,1.06 to 12.48). Compared with patients who had received neoadjuvant chemoradiotherapy, those who received no neoadjuvant treatment had an increased risk of R1 resection (OR 2.82, 1.65 to 4.82). Neoadjuvant chemotherapy without radiotherapy was associated with a higher risk of R1 status than neoadjuvant chemoradiotherapy (OR 2.05, 1.17 to 3.60) (*Table 2*).

**Table 2:** Results of multivariable regression analysis to determine predictors of R1 resection

	Odds ratio	*P*
**Sex**		
F	1.00 (reference)	
M	2.50 (1.36, 4.58)	0.003
**BMI (kg/m^2^)**		
<18.5	2.75 (1.10, 6.83)	0.030
18.5–25	1.47 (0.98, 2.22)	0.062
>25	1.00 (reference)	
**WHO performance score**		
0–1	1.00 (reference)	
>1	1.35 (0.61, 3.01)	0.463
**ASA fitness grade**		
I	1.00 (reference)	
II	1.40 (0.89, 2.22)	0.145
III	1.14 (0.60, 2.17)	0.692
IV	2.89 (0.37, 22.35)	0.309
**Neoadjuvant therapy**		
Chemoradiotherapy	1.00 (reference)	
Chemotherapy	2.05 (1.17, 3.60)	0.012
Radiotherapy	–*	
None	2.82 (1.65, 4.82)	<0.001
**Surgical technique**		
2-field resection	1.00 (reference)	
3-field resection	1.06 (0.54, 2.07)	0.874
Transhiatal	1.46 (0.82, 2.62)	0.199
**Centre caseload (per year)**		
>20	1.00 (reference)	
10–20	0.81 (0.50, 1.32)	0.398
2–10	0.96 (0.54, 1.75)	0.913
<2	1.27 (0.65, 2.50)	0.488
**Clinical tumour category**		
cT1	1.00 (reference)	
cT2	1.18 (0.43, 3.25)	0.751
cT3	2.60 (0.98, 6.90)	0.054
cT4	3.65 (1.06, 12.48)	0.039

Values in parentheses are 95 per cent confidence intervals. *Five observations dropped; predicts failure perfectly.

R1 resection was more common among patients with advanced pathological tumour category: 7 of 221 (3.3 per cent) for pT1, 17 of 336 (5.1 per cent) for pT2, 81 of 603 (13.4 per cent) for pT3, and 33 of 90 (37 per cent) for pT4. Some 184 patients (12.6 per cent) had the pathological finding of either no residual cancer or cancer *in situ*. Twenty-six patients (1.8 per cent) had data missing concerning pathological tumour stage.

### Survival after R1 resection

Median overall survival for the entire cohort was 42 (95 per cent c.i. 38 to 51) months, with a 5-year survival rate of 42.2 (95 per cent c.i. 39.4 to 44.9) per cent. Among patients who had R1 resection, median survival was 20 (16 to 25) months and the 5-year survival rate was 18.0 (11.7 to 25.5) per cent, compared with 50 (41 to 56) months and 44.8 (41.9 to 47.7) per cent for those who had R0 resection.

In the multivariable Cox proportional hazards analysis, R1 resection was an independent risk factor for death within 5 years (HR 1.80, 95 per cent c.i. 1.40 to 2.32). Other factors associated with poor prognosis were male sex (HR 1.29, 1.03 to 1.62), age (60–74 years: HR 1.48, 1.21 to 1.81; 75 years or older: HR 1.74, 1.31 to 2.31), normal BMI (HR 1.22, 1.04 to 1.45), ASA fitness grade III (HR 1.31, 1.01 to 1.71), intermediate level of education (HR 1.30, 1.05 to 1.61), advanced clinical tumour stage (cT3: HR 1.59, 1.05 to 2.37) and clinical node-positive (cN+) disease (HR 1.49, 1.25 to 1.78) (*Table 3*).

**Table 3:** Results of Cox univariable and multivariable regression analyses for all-cause mortality within 5 years

	Univariable analysis		Multivariable analysis	
	Hazard ratio	*P*	Hazard ratio	*P*
**Age (years)**				
<60	1.00 (reference)		1.00 (reference)	
60–74	1.29 (1.09, 1.54)	0.003	1.48 (1.21, 1.81)	<0.001
≥75	1.70 (1.36, 2.12)	<0.001	1.74 (1.31, 2.31)	<0.001
**Sex**				
F	1.00 (reference)		1.00 (reference)	
M	1.40 (1.16, 1.70)	<0.001	1.29 (1.03, 1.62)	0.026
**BMI (kg/m^2^)**				
<18.5	1.53 (1.05, 2.22)	0.025	1.46 (0.92, 2.31)	0.106
18.5–25.0	1.28 (1.10, 1.48)	0.001	1.22 (1.04, 1.45)	0.018
>25.0	1.00 (reference)		1.00 (reference)	
**WHO performance score**				
0–1	1.00 (reference)		1.00 (reference)	
>1	1.58 (1.17, 2.12)	0.003	1.32 (0.91, 1.92)	0.140
**ASA fitness grade**				
I	1.00 (reference)		1.00 (reference)	
II	1.14 (0.97, 1.34)	0.117	1.04 (0.87, 1.26)	0.654
III	1.66 (1.35, 2.06)	<0.001	1.31 (1.01, 1.71)	0.043
IV	2.60 (1.16, 5.84)	0.021	1.19 (0.45, 3.16)	0.720
Level of education (years)*				
Low (≤9)	1.16 (0.95, 1.42)	0.153	1.12 (0.89, 1.41)	0.343
Intermediate (10–12)	1.28 (1.06, 1.55)	0.011	1.30 (1.05, 1.61)	0.016
High (>12)	1.00 (reference)		1.00 (reference)	
**Neoadjuvant therapy**				
Chemoradiotherapy	1.00 (reference)		1.00 (reference)	
Chemotherapy	1.03 (0.85, 1.24)	0.757	0.98 (0.79, 1.21)	0.858
Radiotherapy	0.67 (0.17, 2.68)	0.569	0.63 (0.15, 2.57)	0.524
None	1.18 (1.00, 1.38)	0.049	1.07 (0.85, 1.33)	0.567
**Surgical technique**				
2-field resection	1.00 (reference)			
3-field resection	1.12 (0.89, 1.40)	0.341		
Transhiatal	1.20 (0.95, 1.49)	0.132		
**Year of surgery**				
2012–2017	1.00 (reference)		1.00 (reference)	
2007–2011	1.20 (1.04, 1.38)	0.012	1.14 (0.95, 1.36)	0.152
**Preoperative FDG-PET–CT**				
Yes	1.00 (reference)		1.00 (reference)	
No	1.19 (1.00, 1.41)	0.045	1.20 (0.97, 1.47)	0.087
**Centre caseload (per year)**				
>20	1.00 (reference)			
10–20	0.91 (0.77, 1.07)	0.254		
2–10	1.08 (0.88, 1.32)	0.455		
<2	1.18 (0.91, 1.54)	0.204		
**Microscopic margin**				
R0	1.00 (reference)		1.00 (reference)	
R1	2.04 (1.66, 2.50)	<0.001	1.80 (1.40, 2.32)	<0.001
**Clinical tumour category**				
cT1	1.00 (reference)		1.00 (reference)	
cT2	1.74 (1.20, 2.52)	0.003	1.48 (0.99, 2.21)	0.055
cT3	2.23 (1.55, 3.19)	<0.001	1.59 (1.05, 2.37)	0.025
cT4	1.32 (0.77, 2.26)	0.308	1.03 (0.57, 1.84)	0.921
**Clinical lymph node status**				
Negative	1.00 (reference)		1.00 (reference)	
Positive	1.48 (1.28, 1.71)	<0.001	1.49 (1.25, 1.78)	<0.001
**Tumour location**				
Proximal third oesophagus	1.14 (0.68, 1.92)	0.617	1.42 (0.83, 2.43)	0.199
Middle third oesophagus	1.10 (0.87, 1.38)	0.430	1.05 (0.80, 1.37)	0.714
Lower third oesophagus	1.20 (1 03, 1.41)	0.020	1.17 (0.97, 1.40)	0.095
Oesophagogastric junction	1.00 (reference)		1.00 (reference)	
**Tumour histology**				
Adenocarcinoma	1.00 (reference)			
Squamous cell carcinoma	1.06 (0.88, 1.27)	0.525		

Values in parentheses are 95 per cent confidence intervals.

* Low, corresponds to International Standard Classification of Education (ISCED) level 0–2; intermediate, corresponds to ISCED level 3; high, corresponds to ISCED level 4–6.

## Discussion

R1 resection of oesophageal and oesophagogastric junctional cancer was associated with shortened overall 5-year survival compared with R0 resection. R1 resection was less common among women, those who were overweight, those with less advanced tumours, and among patients who had received neoadjuvant treatment.

The R1 resection rate, generally reported to be in the range 8–19 per cent^4^^,^^11^^,^^12^, was 9.7 per cent in the present study, bearing in mind that this is affected by the classification system used and how meticulously margins are investigated^7^. The circumferential margin was most commonly involved, affecting 7.8 per cent of specimens; this is in the lower range of previous publications based on CAP criteria^7^.

There was a marked difference in R1 frequency between men and women. Men had more than twice the risk of an R1 resection, potentially reflecting different tumour biology or biases in the diagnostic or selection processes between the sexes. Having a low BMI was an independent risk factor for R1 surgery. Preoperative malnutrition has previously been associated with R1 status^4^. Weight loss associated with cancer in the oesophagus and oesophagogastric junction reduces the fatty tissue that embeds the oesophagus, which in obese patients may aid in achieving an R0 resection. In addition, a correlation between malnutrition and poor response to treatment has been established^15^, which could further contribute to this association. Underweight patients are more likely to develop squamous cell carcinoma^16^; however, as histopathology did not affect the frequency of R1 resection in the present study, it is unlikely that the association between being underweight and R1 was mediated through histopathology.

Pathological tumour category (pT) has been found to correlate with R status after oesophagectomy^4^^,^^17^. In this study, cT and cN were used rather than pT and pN, based on the rationale that cT and cN are unaffected by neoadjuvant treatment, available before surgery, and the criteria on which treatment is based. cT was identified as a prognostic factor for R status, although the accuracy of cT may be low^18^.

For pT1–pT2 tumours, 4.3 per cent of resections were R1, and in these patients the involved margins were distributed evenly between longitudinal and circumferential margins. This shows that R1 resection may also occur in low T categories and underlines the importance of adequate margins in oesophagectomy of less advanced tumours. Neoadjuvant therapy decreased the risk of R1 resection^12^^,^^19^, indicative of response in the primary tumour leading to a greater likelihood of having healthy tissue between the tumour and resection margin. As in previous studies^10^^,^^20^, chemoradiotherapy seemed to result in a greater primary tumour response, leading to a higher proportion of R0 resections than among those having neoadjuvant chemotherapy. Although a previous study^4^ did not find neoadjuvant treatment to be an independent predictor of R1 resection, this may simply have been because patients with more advanced tumours are more likely to receive neoadjuvant treatment than those with less advanced tumours.

Overall survival was shorter in patients who underwent R1 compared with R0 resection (median 20 *versus* 50 months), a pattern described by others^11^. Multivariable analysis showed R status to be an independent predictor of survival. To minimize the risk of R1 resection, neoadjuvant treatment should be offered to fit patients. Although neoadjuvant treatment in itself did not affect survival in the present study, the CROSS (ChemoRadiotherapy for Oesophageal cancer followed by Surgery Study) and FLOT4 (Fluorouracil plus Leucovorin, Oxaliplatin and Docetaxel - 4) trials have clearly demonstrated a survival benefit^2^^,21^. In the present study, it is likely that patients with early stages of disease were more likely to have been offered surgery alone, where there was a greater chance of achieving an R0 resection, and this was not fully accounted for by the method of analysis.

Several surgical situations may result in an R1 resection. Locally advanced disease, in which the tumour has grown into the surrounding tissue and fat, may not be taken care of by the inclusion of paraoesophageal tissue and parietal pleura as tumour cells can still be found at the circumferential margin. In patients with T2 disease, where the tumour is confined within the muscle coat of the oesophagus, the resection may still be R1 if the longitudinal margin is inadequate or circumferential R1 if the specimen is damaged during the dissection. Longitudinal microscopic spread of tumour cells from cancer of the oesophagus might be extensive; to ensure R0 resection, a proximal margin of 12 cm^22^ and distal margin of 5 cm^23^ has been recommended for oesophageal cancer. Research on cancer of the oesophagogastric junction suggests a proximal margin of 8 cm to be adequate^24^. Although this margin might be difficult and often impossible to achieve for proximal tumours, the present multivariable analysis did not demonstrate an increased risk of R1 resection for proximal tumours.

The present study involved a large nationwide cohort in a setting in which neoadjuvant treatment with chemoradiotherapy has become the standard of care. To assess R status correctly, it is crucial that the specimens are handled correctly, and the pathologists’ assessments are critical in studies concerning R status. Weaknesses of the study include its retrospective nature. Although data were entered contemporaneously, this might have introduced some selection bias. Incomplete data on some variables was an issue, although nationwide coverage of patients was nearly complete (96 per cent). There may have been some heterogeneity in preoperative staging and pathological assessment reflecting local centre policies, although national guidelines were in place and generally used. For some variables, data from relatively few patients were included in the survival analysis, for example being underweight, having an ASA grade of IV, and cT4 classification. It is acknowledged that other studies^25^^,^^26^ have shown that these features influence survival.

## Funding

M.L. received a grant from the Swedish Cancer Society (180787).

*Disclosure.* The authors declare no conflict of interest.

## Notes

Presented to the 2019 Annual Meeting of the European Society for Diseases of the Esophagus, in Athens, Greece, November 2019. https://doi.org/10.1093/dote/doz092.30.
